# International study to develop a patient-reported outcome measure to evaluate outcomes of gender-affirming care - the GENDER-Q

**DOI:** 10.1186/s41687-024-00785-x

**Published:** 2024-11-19

**Authors:** Manraj N. Kaur, Shane D. Morrison, Shelby L. Kennedy, Tim C. van de Grift, Astrid Højgaard, Amalie Lind Jacobsen, Natasha Johnson, Margriet G. Mullender, Lotte Poulsen, Thomas Satterwhite, Richard Santucci, John Semple, Charlene Rae, Kinusan Savard, Jens Ahm Sørensen, Danny Young-Afat, Andrea L. Pusic, Anne F. Klassen

**Affiliations:** 1 Brigham and Women’s Hospital, Harvard Medical School, 75 Francis S, Boston, MA 02116 USA; 2 Division of Plastic Surgery, Department of Surgery, University of Washington, 325 9th Ave, Mail Stop #359796, Seattle, WA 98104 USA; 3 Department of Pediatrics, McMaster University, 1280 Main Street W, Hamilton, ON L8N 3Z5 Canada; 4 Department of Plastic Reconstructive and Hand Surgery, Center of Expertise on Gender Dysphoria, Amsterdam Public Health Research Institute, VU University Medical Center, De Boelelaan 1117, Amsterdam, 1081 HV The Netherlands; 5 Sexological Center, Center for Gender, Center for Rape Victims, Aalborg University Hospital, Aalborg, Denmark; 6 Research Unit for Plastic Surgery, University of Southern Denmark and Odense, Denmark and OPEN, Odense Explorative Patient Network, Odense, Denmark; 7 Align Surgical Associates Inc., 2299 Post St. Suite 207, San Francisco, CA 94115 USA; 8 Crane Center, 4407 Bee Caves Rd. Ste. 612, Austin, TX 78746 USA; 9 Division of Plastic, Reconstructive and Aesthetic Surgery, University of Toronto, The Rotman/Stewart Building, 149 College Street, 5th Floor, Suite 508, Toronto, ON M5T 1P5 Canada; 10 Fleming College - Emeritus, RR1, Noelville, ON P0M 2N0 Canada

**Keywords:** Gender-affirming care, Transgender, Gender diverse, Patient-reported outcome measure, Patient-reported outcomes, Top surgery, Bottom surgery, Facial feminization, Nonbinary, Gender-affirming surgery

## Abstract

**Background:**

To meaningfully understand outcomes of gender-affirming care, patient-reported outcome measures (PROMs) that are grounded in what matters to individuals seeking care are urgently needed. The objective of this study was to develop a comprehensive PROM to assess outcomes of gender-affirming care in clinical practice, research, and quality initiatives (the GENDER-Q).

**Methods:**

Internationally established guidelines for PROM development were used to create a field test version of the GENDER-Q. In-depth interviews were conducted from December 2018 to March 2020 with youth and adults aged 16 years and older who were seeking or had received gender-affirming care at outpatient clinics providing gender-affirming care located within tertiary care centers or communities in Canada, Denmark, the Netherlands or the US. Data were analyzed and used to develop a conceptual framework and an item pool, which was used to develop preliminary scales. Between February 2021 to November 2021, iterative feedback was sought from clinicians and patient participants on the scales and used to refine or develop new scales. The revised scales were pilot-tested using a crowd-sourcing platform between February 2022 and April 2022.

**Results:**

Data from interviews with 84 participants (aged 34 ± 14 years) resulted in a conceptual framework of the GENDER-Q with 13 domains measuring health-related quality of life, sexual, urination, gender practices, voice, hair, face and neck, body, breasts, genital feminization, chest, genital masculinization, and experience of care. Preliminary versions of 44 scales were developed covering most concepts in the conceptual framework. Iterative feedback was obtained from clinician experts (4 to 37 experts per scale; response rate, 67%) and 7–14 patient participants (depending on scale). All scales were refined, and 15 new scales were developed, resulting in 55 scales in the field test version of the GENDER-Q. In total, 601 transgender and gender diverse (TGD) people (aged 25 ± 6 years) participated in the pilot field test and the data were used to make changes to the field test survey.

**Conclusion:**

The GENDER-Q was developed using extensive input from TGD individuals and clinician experts and represents the most comprehensive set of independently functioning scales that are available to date. An international field test of the GENDER-Q was completed in 2024 and the GENDER-Q is available for use in patient care, clinical research and quality improvement efforts.

**Supplementary Information:**

The online version contains supplementary material available at 10.1186/s41687-024-00785-x.

## Background

Transgender and gender diverse (TGD) people are those whose gender identities or expressions differ from their sex assigned at birth [1, 2]. Gender affirmation refers to the process of recognizing or affirming TGD people in their gender identity or expression—socially, medically, legally, behaviourally, or a combination of these [1, 2]. Gender-affirming care is medically necessary, complex, and individualized. The person-centred nature of gender-affirming care underscores the importance of understanding the individual beyond their clinical presentation, including, their symptoms, functional status, psychosocial distress, sexual well-being, treatment goals, and experiences of healthcare. This approach is well-suited for the measurement of patient-reported outcomes.

Patient-reported outcomes (PROs) are outcomes that are reported directly by a patient without any interpretation of their response by a clinician or anyone else [3]. Fundamentally, patient-reported outcome measures (PROMs) ensure that the patient’s voice is meaningfully captured in treatment-decision making and comparative effectiveness research. However, no rigorously developed and validated gender-affirming care-specific PROM currently exists. Results from five recent systematic reviews on PROMs in TGD research convergently conclude that the PROMs used in TGD research are either generic, designed for cisgender populations, or were not developed following internationally recommended guidelines for the development of PROMs [4–8]. When PROMs that do not have content validity (i.e., relevant, comprehensive, and comprehensible content) in the context of gender-affirming care are used, they fail to measure what matters to patients, and hence, cannot be used to capture the voices of TGD individuals in clinical care and research.

A PROM that is rigorously developed, validated and covers the full range of PROs relevant to gender-affirming treatments and services is urgently needed. The aim of our international study was to develop and refine the content of a comprehensive, modular PROM called the GENDER-Q for use in clinical care, clinical research, quality improvement initiatives and regulatory efforts to evaluate the PROs associated with gender-affirming interventions.

## Methods and analysis

**Fig. 1:**
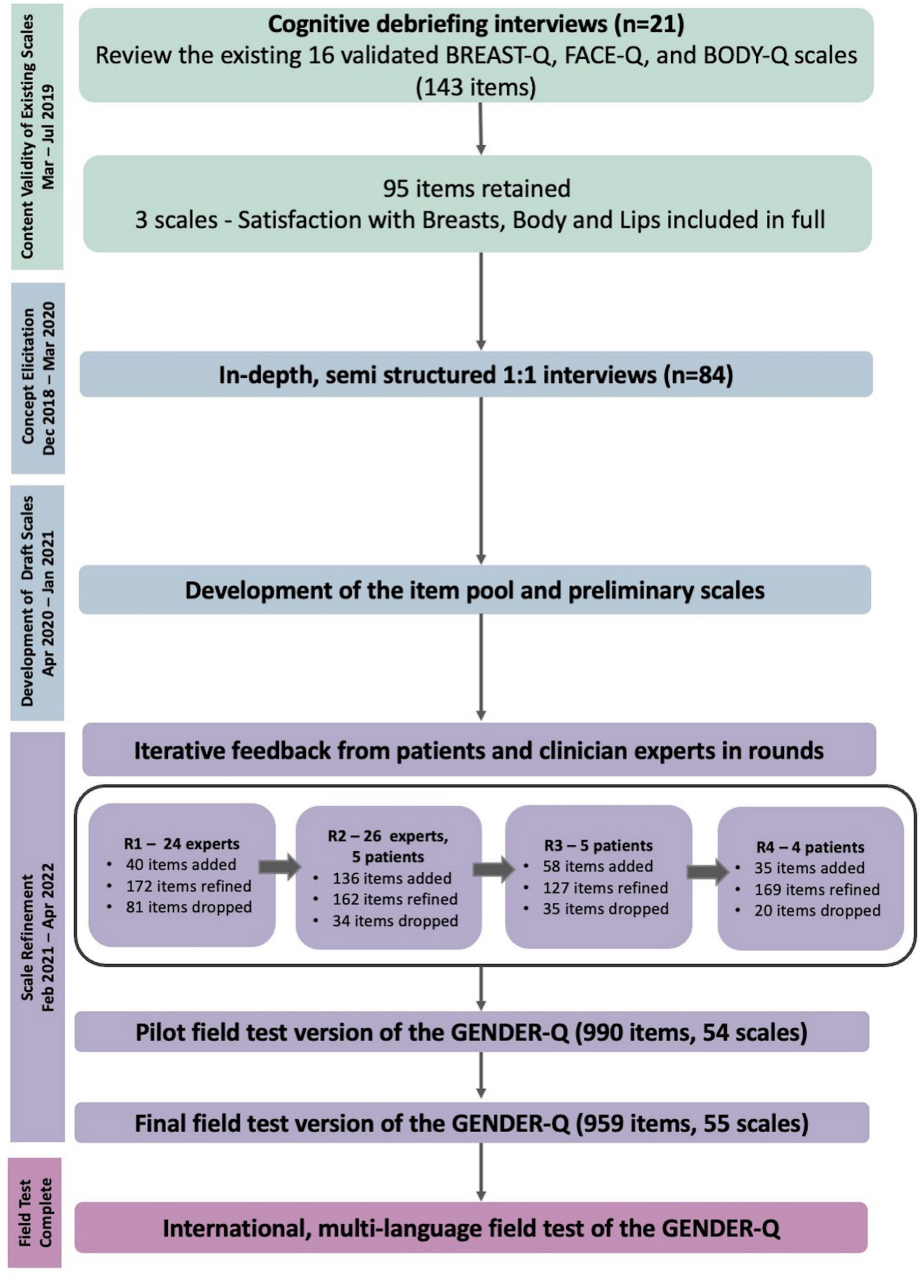
Overview of the multi-step approach used for the development of the GENDER-Q

A protocol paper describing the development of the GENDER-Q has been previously published [9]. The development of GENDER-Q follows internationally established guidelines for PROM development [3, 10–12] and consists of two main steps: (1) development of a field test version of the GENDER-Q, and (2) psychometric evaluation of the GENDER-Q. This paper describes step 1 of the GENDER-Q development. Figure 1 provides an overview of the development of the GENDER-Q. Research ethics board approval was obtained from the Hamilton Integrated Research Ethics Board (Canada; coordinating site), the Medical Ethical Committee at Amsterdam University Medical Center, VUmc (The Netherlands) and Advarra (United States (US)). In Denmark, the study was included on the list of health research (exempt) within the Region of Southern Denmark.

**Fig. 2:**
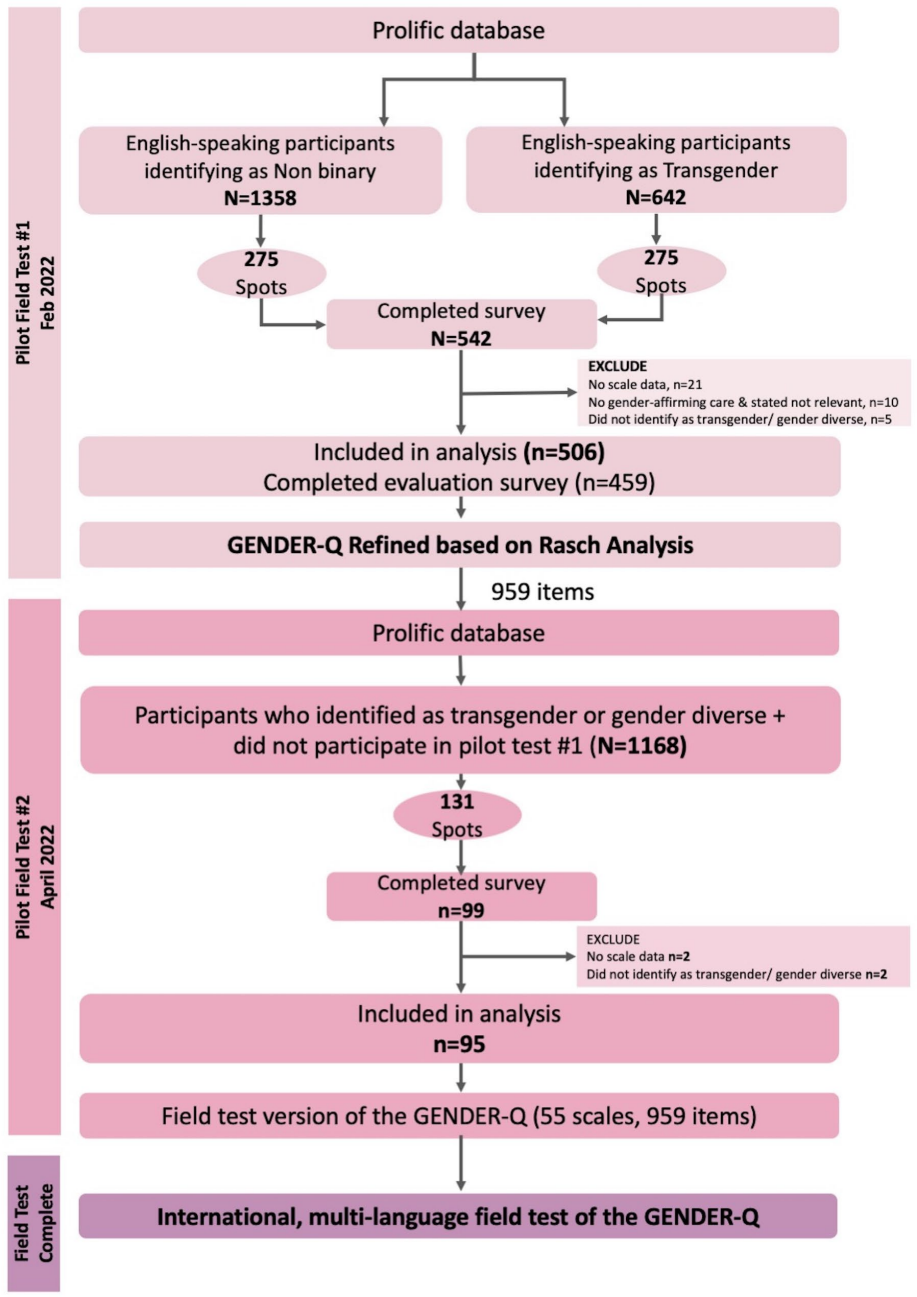
Overview of the pilot field test of the GENDER-Q

### Sample and recruitment

Individuals who identified as TGD, were 16 years or older, seeking or had received gender-affirming care, able to provide informed consent and fluent in English (Canada, the Netherlands, the US), Danish (Denmark) or Dutch (the Netherlands) were recruited from six specialized high-volume centers that provide gender-affirming care in Canada, Denmark, the Netherlands, and the US. Purposive sampling was used to recruit a sample varied by age, gender, ethnicity, type of gender-affirming treatment, and time since treatment. Eligible participants were informed of the study at their in-person routine clinic appointment, by telephone, or by email and the contact information for interested patients was shared with the site research coordinator. In the Netherlands, participants were also recruited through the TGD community groups. The study information sheet was reviewed with the potential participants and written and verbal consent was obtained. Interviews took place by phone (Canada, US) or in person—in a private clinic space (Canada, Denmark, the Netherlands) or at the participant’s home (the Netherlands). Participants from Canada, the Netherlands, and the US were provided monetary compensation for their participation.

See supplementary materials for interview guides for part 1, 2 and 3. All the interviews were audio-recorded and transcribed verbatim with identifying information removed.

### Part 1: evaluating content validity of existing scales

Cognitive debriefing interviews using the “think aloud” technique [13] were conducted with English-speaking patient participants from Canada and the US to obtain feedback on a subset of 12 scales that measure satisfaction with the body, breast, and face, and 4 scales that measure patients’ experience of care from the BREAST-Q [14], FACE-Q [15], and BODY-Q [16]. These scales were hypothesized to contain relevant content (i.e., items) for gender-affirming care (see Supplementary Material 1 for interview guide). For analysis, codes related to items from the BREAST-Q, FACE-Q and BODY-Q were organized by scale and item number and examined to identify content deemed relevant to gender-affirming care. Relevant items were included in the GENDER-Q scales.

### Part 2: concept elicitation through in-depth interviews

In-depth semi-structured interviews were conducted whereby the participants were asked to describe their treatment history and any planned or future treatments. Following this, participants were asked to share the impact of gender-affirming care on their health-related quality of life (HRQL) (e.g., appearance, body image, voice, psychosocial well-being) and satisfaction with experience of care and pre-operative information (for surgical patients only) (see Supplementary Material 1 for interview guide). Demographic and clinical information was collected on a pre-set form. The part 2 interviews in Danish and Dutch were translated into English and coded alongside the English transcripts using a line-by-line approach. Codes were transferred into Excel for further analysis. Constant comparison was used to identify the top-level domains and subdomains, which led to the development of a conceptual framework covering outcomes important to people who were seeking or have had gender-affirming care. The analysis also led to the development of an item pool for use in GENDER-Q scale development. Specifically, for each important concept of interest, items from parts 1 and 2 were used to map out the concept to form a scale, and instructions, recall period, and response options were drafted. The ordering of items followed a modern psychometric approach - the Rasch Measurement Theory (RMT) - to scale design, ensuring that each scale measures a clinical hierarchy [17].

To ensure rigor, the concepts elicited during the interviews were confirmed iteratively throughout the course of interviews. Additionally, the first set of 10 interviews from each country were either double-coded by two team members who then met to review codes, or coded by one member with the codes subsequently reviewed by a second member. The study team met regularly throughout the study to review the codebook and emerging concepts.

### Part 3: scale refinement

Scales were refined iteratively through multiple rounds of feedback from patient participants and clinical and research experts in gender-affirming care. Clinical experts known to the GENDER-Q team were invited via email to review the GENDER-Q scales and provide feedback. Given the large number GENDER-Q scales, experts were invited to review as many as they were able to or to focus on the subset that fell within their area of expertise. Experts were asked to use track changes or the comment feature in Microsoft Word to provide feedback on the comprehensibility, comprehensiveness and relevance of scale instructions, recall period, response options and items, and to suggest missing items.

English-speaking patient participants from the US and Canada who took part in part 2—concept elicitation interviews and new patients were invited to review the GENDER-Q scales in cognitive debriefing interviews using the think-aloud technique [13]. The interviews were conducted in three rounds by phone or an institutionally licensed virtual conferencing platform (i.e., Zoom). The patient participants were asked to comment on scale’s instructions, recall period, response options and items to ensure that the scales were easy to understand and relevant (see Supplementary Material 1 for interview guide). At the end of the interview, participants were asked if the scales and the GENDER-Q conceptual framework were comprehensive and to nominate missing items or scales [10–12]. The interviews were coded line-by-line and analyzed. Expert and patient participant input was used to iteratively refine the GENDER-Q scales until no more changes were deemed necessary.

### Part 4: pilot field test

A pilot field test of the GENDER-Q scales was conducted in 2 parts using an online crowdsourcing research platform called Prolific (https://www.prolific.co) (Fig. 2). Prolific members who were 18 years or older, identified as transgender or nonbinary and were fluent in English (any country) were sent a link to an online REDCap survey. Participants self-selected as eligible and provided consent before completing clinical and demographic questions and the GENDER-Q scales. Branching logic was used to ensure participants only answered scales relevant to their experience. An open-ended text box was included for additional feedback. Participants were compensated at a prorated hourly rate of $18 USD.

Exploratory Rasch Measurement Theory (RMT) analysis was performed to examine scale performance using RUMM2030 software with the unrestricted Rasch model for polytomous scales (RUMM version 2030, RUMM Laboratory Pty Ltd, Duncraig, Western Australia, Australia, 1998–2023). The analysis examined the fit of items to the Rasch model. Items with extreme misfit to the Rasch model were removed. Items were reordered according to the item location order for each scale (i.e., clinical hierarchy). Open-text comments were reviewed for feedback on questions or branching logic, and the survey was updated accordingly. A second and final pilot field test was conducted with the updated version of the GENDER-Q to ensure the functionality of the survey, resulting in the final field test version of the GENDER-Q.

## Results

### Part 1: evaluating content validity of existing scales

Cognitive debriefing interviews (*n* = 21) took place between March and July 2019. Table 1 shows the characteristics of the sample. Overall, much of the content from the existing scales resonated with participants. Table 2 shows the existing PROM scales that were included in the GENDER-Q. Of the 16 scales reviewed, 95 of the 143 items were considered relevant and covered important issues for people undergoing breast/chest surgery, body contouring and facial feminization. A further eight items were added to address missing concepts suggested by participants during the scale refinement phase (e.g., “how attractive your cheeks look?” from the FACE-Q Cheeks scale). The item set for three scales measuring satisfaction with breasts, body and lips were included in full.

**Table 1:** Characteristics of participants in Step 1 of GENDER-Q development

		Content generation		Part 3 Scale refinement (*n* = 14) N (%)
		Part 1 Content validity of existing scales(*n* = 21) N (%)	Part 2 Concept elicitation (*n* = 84) N (%)	
Country	Canada	5 (24)	20 (24)	1 (7)
	Denmark	0	12 (14)	0
	The Netherlands	0	21 (25)	0
	United States	16 (76)	31 (37)	13 (93)
Gender identity	Trans masculine	3 (14)	42 (50)	7 (50)
	Trans feminine	18 (86)	37 (44)	7 (50)
	Nonbinary / Gender queer/ Gender non-conforming	0	5 (6)	0
Age	16–19	0	14 (17)	0
	20–29	4 (19)	23 (27)	0
	30–39	9 (43)	20 (24)	8 (57)
	40–49	5 (24)	11 (13)	2 (14)
	50–59	2 (10)	11 (13)	3 (21)
	*≥* 60	1 (5)	5 (6)	1 (7)
Race	White	16 (76)	53 (63)	12 (86)
	Other	4 (19)	9 (11)	2 (14)
	Prefer to not answer/missing	1 (5)	22 (26)	0
Marital status	Single, never married	5 (24)	35 (42)	3 (21)
	Married / Living common law	5 (24)	20 (24)	7 (5)
	Divorced/Separated/Not in relationship	3 (14)	10 (12)	1 (7)
	Currently in relationship	8 (38)	19 (23)	3 (21)
Education	Some high school/Completed high school	2 (10)	32 (38)	0
	Some college, trade or university	4 (19)	8 (10)	3 (21)
	Completed college, trade or university	12 (57)	36 (43)	8 (57)
	Completed Masters/Doctoral degree	3 (14)	8 (10)	3 (21)
Treatment type	Masculinization	3 (14)	47 (56)	7 (50)
	Feminization	18 (86)	37 (44)	7 (50)
Reported having	Voice surgery and/or therapy (all)	6 (29)	15 (18)	3 (21)
	Body contouring	1 (6)	1 (1)	1 (7)
	*Feminizing procedures*			
	Tracheal shave (feminization participants only)	1 (6)	3 (8)	0
	Facial feminization surgery	3 (17)	6 (16)	1 (14)
	Surgery to augment the chest	8 (44)	10 (27)	2 (29)
	Vaginoplasty	16 (89)	22 (59)	6 (86)
	*Masculinizing procedures*			
	Surgery to flatten or contour the chest	3 (100)	31 (83)	7 (50)
	Phalloplasty	0	10 (21)	5 (71)
	Metoidioplasty	0	6 (13)	1 (14)
	Scrotoplasty	0	5 (11)	2 (29)
	Glansplasty	0	5 (11)	2 (29)
	Erectile device	0	3 (6)	1 (14)

**Table 2:** Scales from the BREAST-Q, FACE-Q and BODY-Q reviewed by participants and summary of change in number of items after participant feedback

PROM	Scales	No. of items in the scale	No. of items after Part 1	No. of items in the pilot field test version	Example item
BREAST-Q	Breasts	15	15	15	How your bras fit?
FACE-Q	Face overall	10	5	5	How your face looks in photos?
	Forehead & Eyebrows	6	1	3	The position of your eyebrows?
	Eyes	7	2	2	How open your eyes look?
	Lips	10	10	10	How full your lower lip looks?
	Chin	10	8	8	The size of your chin?
	Cheeks	5	3	4	How attractive your cheeks look?
	Cheekbones	10	0	2	How high your cheekbones look?
	Nose	10	9	9	The overall size of your nose?
	Nostrils	5	2	4	The shape of your nostrils?
	Lower Face & Jawline	5	2	3	The shape of your jawline?
BODY-Q	Body	10	10	10	How your clothes fit your body?
	Information	10	4	4	How the surgery would be done?
	Surgeon	10	9	9	Spent enough time with you?
	Medical team	10	8	8	Treated you with respect?
	Office staff	10	7	7	Answered all your questions?
Total		143	95	103	

### Part 2: concept elicitation through in-depth interviews

A total of 85 participants were interviewed between December 2018 and March 2020. One participant subsequently withdrew from the study. Table 1 shows the characteristics of the 84 participants in the study sample. More than half of the participants were seeking or had undergone masculinization treatments. The most common procedure was chest surgery, and the least common procedures were body contouring and tracheal shaving. The analysis led to the development of a preliminary framework of concepts that are important to measure when assessing outcomes of gender-affirming care.

### Part 3: scale refinement

Important concepts from the qualitative data, supplemented by relevant items from the BREAST-Q, FACE-Q and BODY-Q scales from part 1, were used to develop version 1 of the GENDER-Q. For surgical scars (chest, donor site), concepts from the qualitative data that overlapped with the SCAR-Q developed by our team were used [18, 19]. Additionally, the Animation Deformity scale from the BREAST-Q that measures the distortion in the chest appearance with the contraction of the pectoralis muscle for individuals with chest augmentation was included [20].

The GENDER-Q scales were refined between February and November 2021. We obtained feedback from 50 of 75 invited experts (response rate, 67%). The experts were from the US (*n* = 28), Denmark (*n* = 6), Canada (*n* = 5), The Netherlands (*n* = 4), Belgium (*n* = 3), Spain (*n* = 3), and Switzerland (*n* = 1). Experts’ specialties included plastic surgery (*n* = 30), psychology (*n* = 4), urology (*n* = 3), endocrinology (*n* = 3), speech therapy (*n* = 3), research (*n* = 3), gynecology (*n* = 2), and others (*n* = 2). Depending on the scale, feedback was obtained from 4 to 37 experts and 7–14 patient participants (See Supplementary Table 2). All but one patient participant in the scale review had previously participated in the part 2 concept elicitation interview. Table 3 summarizes changes made to the GENDER-Q scales between the rounds. Items were added or deleted due to issues with comprehension including participants not interpreting key concepts and questions as intended, requesting clarification on the meaning of specific words or entire item, or experiencing difficulty with recall or judgement. Other reasons for refining or deleting items included challenges in forming responses, indications that an item was overtly sensitive or caused gender dysphoria, age or gender-related nuances that could influence interpretation or response to an item, and participants identifying items as being too long or difficult to understand.

**Table 3:** Changes made to GENDER-Q scales in each round of patient and expert feedback in Part 3 and pilot field test in Part 4

Domain	GENDER-Q Scales	V1	Round 1				V2	Round 2			
			Retain	Revise	Drop	Add		Retain	Revise	Drop	Add
HRQL	Body Image	15	0	8	7	3	11	10	1	0	2
	Gender Dysphoria	16	0	16	0	1	17	17	0	0	0
	Social Acceptance	24	20	4	0	1	25	19	4	2	0
	Psychological Distress	20	20	0	0	0	20	19	1	0	1
	Psychological Well-Being	21	19	0	2	0	19	19	0	0	0
	Treatment Outcome	22	9	10	3	0	19	15	2	2	1
Sexual	Sexual Well-Being	21	8	9	4	2	19	8	6	5	1
	Orgasm	–	–	–	–	–	–	–	–	–	–
Urination	Urination	19	18	1	0	1	20	13	6	1	3
	Urinary Catheter	16	14	1	1	0	15	14	1	0	1
Gender Practices	Binding Well-Being	–	–	–	–	–	13	12	1	0	0
	Binding: Adverse Effects	25	18	2	5	0	20	19	1	0	0
	Tucking: Adverse Effects	15	6	5	4	2	13	13	0	0	2
Voice	Sound	16	7	7	2	3	17	14	3	0	4
	Distress	14	10	4	0	0	14	11	2	1	2
Hair	Hair – Face^$^	14	12	0	2	2	14	12	1	1	2
	Hair – Face	–	–	–	–	–	13	7	4	2	5
	Hair - Head	14	10	2	2	0	12	11	1	0	3
	Hair – Body	13	0	0	0	0	0	–	–	–	–
Face & Neck	Face	19	18	1	0	3	22	21	1	0	3
	Upper Face	18	12	2	4	0	14	11	3	0	1
	Nose	17	14	2	1	0	16	7	9	0	4
	Nostrils	7	6	1	0	0	7	5	2	0	2
	Lips	20	16	2	2	1	19	17	2	0	3
	Cheeks**	–	–	–	–	–	10	8	2	0	3
	Chin	14	10	2	2	0	12	10	1	1	3
	Jawline	12	9	2	1	0	11	6	4	1	4
	Adam’s Apple	12	10	1	1	0	11	10	0	1	4
Body	Body**	–	–	–	–	–	11	10	1	0	1
	Buttocks**	–	–	–	–	–	7	6	1	0	3
	Waist**	–	–	–	–	–	8	7	1	0	1
Breasts	Breasts	21	18	3	0	0	21	19	2	0	1
	Nipples Areolas	11	2	8	1	2	12	9	3	0	1
	Animation Deformity**	–	–	–	–	–	12	12	0	0	0
Genital Feminization	Vagina	17	13	4	0	1	18	12	5	1	4
	Labia	30	25	3	2	1	29	12	16	1	0
	Clitoris	16	9	5	2	0	14	6	2	6	8
Chest	Chest	21	1	14	6	1	16	13	3	0	1
	Nipples/areolas	15	4	8	3	0	12	6	6	0	1
	Scars**	–	–	–	–	–	12	12	0	0	6
Genital Masculinization	Penis	24	16	6	2	4	26	12	13	1	5
	Penis Sensation	10	10	0	0	2	12	12	0	0	5
	Glans	15	8	3	4	2	13	0	12	1	1
	Scrotum	22	15	3	4	0	18	10	8	0	6
	Perineum	12	7	3	2	1	11	2	9	0	2
	Phalloplasty Flap	14	14	0	0	0	14	8	5	1	1
	Phalloplasty Scars**	–	–	–	–	–	12	12	0	0	5
	Donor Site: Adverse Effects	10	10	0	0	1	11	9	2	0	2
	Testicular implants	11	9	2	0	1	12	4	8	0	3
	Erectile device	17	3	13	1	0	16	6	5	5	8
Experience of care	Healthcare Professional	40	30	3	7	2	35	35	0	0	2
	Clinic	21	12	6	3	1	19	18	0	1	1
	Surgery - Information	20	13	6	1	0	19	19	0	0	5
	Surgery - Adverse Effects	25	25	0	0	1	26	26	0	0	6
	Surgery - Return to Activity	16	16	0	0	1	17	15	2	0	3

Domain	GENDER-Q Scales	V3	Round 3				V4	Round 4				Pilot FT	Pilot FT	
			Retain	Revise	Drop	Add		Retain	Revise	Drop	Add		Drop	Revise
HRQL	Body Image	13	12	1	0	1	14	13	1	0	0	14		
	Gender Dysphoria	17	14	3	0	1	18	0	18	0	0	18	2	
	Social Acceptance	23	15	6	2	1	22	21	0	1	0	21	1	
	Psychological Distress	21	21	0	0	1	22	20	0	2	0	20	1	
	Psychological Well-Being	19	19	0	0	2	21	21	0	0	0	21		
	Treatment Outcome	18	16	1	1	0	17	16	0	1	0	16		
Sexual	Sexual Well-Being	15	12	2	1	3	17	16	1	0	1	18	2	
	Orgasm	14	11	3	0	1	15	0	11	4	4	15	1	
Urination	Urination	22	16	4	2	5	25	18	6	1	1	25		
	Urinary Catheter	16	9	7	0	0	16	11	4	1	0	15	NA	NA
Gender Practices	Binding Well-Being	13	–	–	–	–	13	7	5	1	0	12		
	Binding: Adverse Effects	20	–	–	–	–	20	20	0	0	1	21		
	Tucking: Adverse Effects	15	10	3	2	2	15	14	1	0	0	15		
Voice	Sound	21	18	2	1	1	21	17	3	1	2	22	3	
	Distress	15	13	1	1	0	14	11	2	1	1	14	1	
Hair	Hair – Face^$^	15	11	3	1	3	17	11	6	0	1	18		Merged into 1 scale
	Hair – Face	16	–	–	–	–	16	16	0	0	1	17		
	Hair - Head	15	9	4	2	4	17	14	3	0	1	18		
	Hair – Body	–	–	–	–	–	–	–	–	–	–	–	–	Scale dropped
Face & Neck	Face	25	23	2	0	3	28	26	2	0	2	30		Split into 2 scales
	Upper Face	15	8	6	1	7	21	19	1	1	1	21		Split into 2 scales
	Nose	20	14	4	2	0	18	13	4	1	1	18		
	Nostrils	9	6	1	2	2	9	6	3	0	1	10		
	Lips	22	18	4	0	2	24	16	8	0	0	24		
	Cheeks**	13	10	3	0	2	15	15	0	0	0	15		
	Chin	14	11	3	0	0	14	12	2	0	0	14	1	
	Jawline	14	10	3	1	1	14	13	1	0	0	14	2	
	Adam’s Apple	14	10	2	2	2	14	13	1	0	2	16		
Body	Body**	12	10	2	0	0	12	12	0	0	0	12		
	Buttocks**	10	9	1	0	0	10	9	1	0	1	11		
	Waist**	9	7	1	1	0	8	7	1	0	0	8		
Breasts	Breasts	22	19	3	0	0	22	21	1	0	1	23		
	Nipples Areolas	13	8	3	2	2	13	13	0	0	0	13		
	Animation Deformity**	12	12	0	0	0	12	12	0	0	0	12	NA	NA
Genital Feminization	Vagina	21	16	3	2	0	19	16	3	0	2	21	NA	NA
	Labia	28	25	2	1	1	28	24	3	1	1	28	NA	NA
	Clitoris	16	12	3	1	2	17	14	3	0	0	17	NA	NA
Chest	Chest	17	–	–	–	–	17	16	1	0	0	17		
	Nipples/areolas	13	9	3	1	2	14	12	2	0	1	15		
	Scars**	18	–	–	–	–	18	17	1	0	1	19		
Genital Masculinization	Penis	30	–	–	–	–	30	21	9	0	3	33	NA	NA
	Penis Sensation	17	–	–	–	–	17	0	15	2	1	16	NA	NA
	Glans	13	–	–	–	–	13	9	4	0	2	15	NA	NA
	Scrotum	24	–	–	–	–	24	19	5	0	1	25	NA	NA
	Perineum	13	10	3	0	2	15	10	4	1	0	14	NA	NA
	Phalloplasty Flap	14	–	–	–	–	14	13	1	0	0	14	NA	NA
	Phalloplasty Scars**	17	–	–	–	–	17	17	0	0	1	18	NA	NA
	Donor Site: Adverse Effects	13	–	–	–	–	13	12	1	0	0	13	NA	NA
	Testicular implants	15	–	–	–	–	15	10	4	1	0	14	NA	NA
	Erectile device	19	–	–	–	–	19	15	4	0	0	19	NA	NA
Experience of care	Healthcare Professional	37	29	6	2	0	35	32	3	0	0	35	2	
	Clinic	19	3	16	0	1	20	17	3	0	0	20	1	
	Surgery - Information	24	12	11	1	3	26	18	8	0	0	26	NA	NA
	Surgery - Adverse Effects	32	28	1	3	1	30	25	5	0	0	30		
	Surgery - Return to Activity	20	19	1	0	0	20	16	4	0	0	20	NA	NA

*FT* field test, *NA* not applicable due to the insufficient sample size of the pilot field test sample

**Represents existing BREAST-Q, FACE-Q, BODY-Q or SCAR-Q scales

^$^Feminization version of the scale

### Part 4: pilot field test

A total of 601 participants from 30 countries were included in the pilot field test (parts 1 and 2) (Fig. 2). Sample demographics for the pilot field tests are shown in Table 4.

**Table 4:** Demographic characteristics of the pilot field test sample

		Pilot field test 1		Pilot field test 2		Total	
		*N* = 506		*N* = 95		*N* = 601	
		n	%	n	%	n	%
Gender identity (best describes)	Trans masculine	137	27	56	59	193	32
	Trans feminine	81	16	15	16	96	16
	Nonbinary	258	51	19	20	277	46
	Other (e.g., gender fluid)	30	6	5	5	35	6
Sex assigned on original birth certificate	Male	145	29	22	23	167	28
	Female	361	71	73	77	434	72
Age	18–19 years	60	12	18	19	78	13
	20–29 years	365	72	59	62	424	71
	30–39 years	67	13	14	15	81	13
	40 years or more	14	3	4	4	18	3
Race	White	408	81	75	79	483	80
	Latin American (e.g., Mexico, Central America, Caribbean islands)	46	9	6	6	52	9
	Black (e.g., African, Caribbean, African Canadian/American descent)	25	5	2	2	27	4
	Other	27	5	12	13	39	6
Highest level of education attained	Some high school	18	4	3	3	21	3
	Completed high school	98	19	25	26	123	20
	Some college or trade school or university	203	40	33	35	236	39
	Completed college or trade school or university degree	132	26	23	24	155	26
	Some Masters or Doctoral degree	22	4	4	4	26	4
	Completed Masters or Doctoral degree	31	6	6	6	37	6
	Prefer not to answer	2	0	1	1	3	0
Country of residence	United States	145	29	29	31	174	29
	United Kingdom	104	21	29	31	133	22
	Poland	41	8	5	5	46	8
	Mexico	31	6	2	2	33	5
	Canada	23	5	3	3	26	4
	Italy	22	4	0	0	22	4
	Portugal	19	4	7	7	26	4
	South Africa	19	4	3	3	22	4
	Spain	19	4	2	2	21	3
	Germany	14	3	4	4	18	3
	Netherlands	10	2	0	0	10	2
	Other	59	12	11	12	70	12
Reported having	Hormones (currently)	185	37	51	54	236	39
	Hair transplant (head)	3	1	0	0	3	0
	Scalp Advancement	1	0	1	1	2	0
	Facial surgery	11	2	3	3	14	2
	Facial hair removal (feminization participants only)^*^	26	20	6	35	32	22
	Voice therapy	48	10	7	7	55	9
	Voice surgery	1	0	0	0	1	0
	Tracheal shave (feminization participants only)^@^	4	3	1	5	5	3
	Surgery to flatten or contour the chest (masculinization participants only)^#^	60	20	17	24	77	21
	Surgery to augment the chest (feminization participants only)^^^	7	5	1	4	8	5
	Surgery to create genitals	9	2	2	2	11	2
	Body contouring	5	1	1	1	6	1

^*^Pilot field test 1, *n* = 129; Pilot field test 2, *n* = 17

^@^Pilot field test 1, *n* = 145; Pilot field test 2, *n* = 22

^#^Pilot field test 1, *n* = 296; Pilot field test 2, *n* = 70

^^^Pilot field test 1, *n* = 138; Pilot field test 2, *n* = 25)

**Fig. 3:**
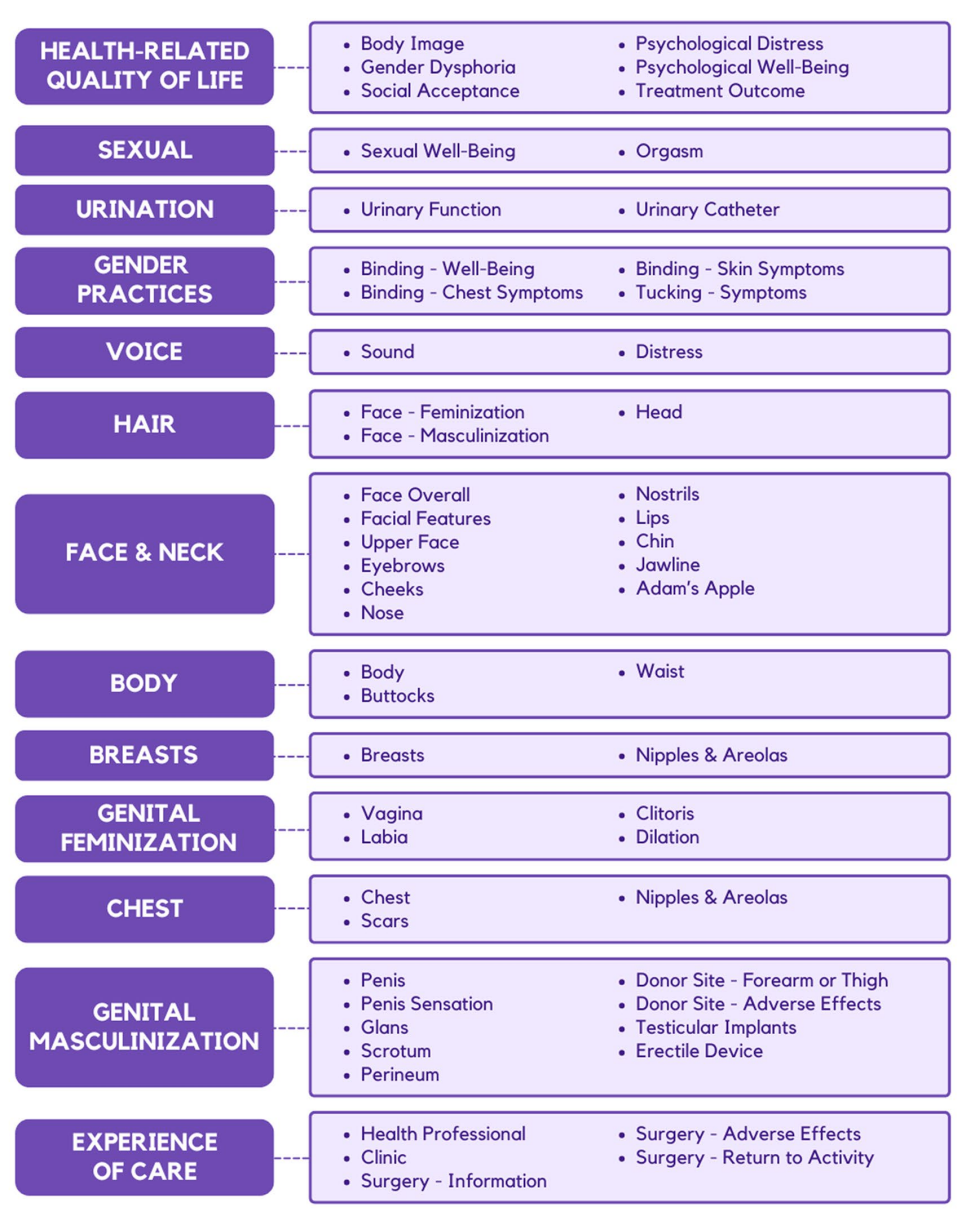
Conceptual Framework of the GENDER-Q

Based on the exploratory RMT analysis of the part 1 pilot field test data, 17 items with poor item fit in 11 scales were removed. The Appearance—Face scale was divided into two scales that measured the appearance of the face and the appearance of facial parts. The Appearance—Upper Face scale was split to measure the appearance of the upper face and the appearance of eyebrows. The masculine and feminine Appearance—Facial Hair scales were modified to create a single satisfaction scale that was applicable across the gender spectrum. Seventeen GENDER-Q scales (all genital surgery-related, Return to Activity, Catheter, and Breast—Animation Deformity, and Information) did not have sufficient data for RMT analysis and were not altered. The items in all remaining scales were reordered according to the clinical hierarchy based on the item locations from the RMT analysis. Figure 3 shows the conceptual framework for GENDER-Q. Each component of the framework corresponds to an independently functioning GENDER-Q scale.

Several iterative changes were made to the GENDER-Q survey based on participants’ open-text comments. First, at the start of the survey, a box was added with the following content warning: “The GENDER-Q asks detailed questions about how you look and feel, and about gender-affirming care. Some people have said that some parts of the GENDER-Q made them feel uncomfortable or upset. If you take part in our study, most questions are set up to allow you to skip them if you do not want to answer. Some questions are required as these are used to make sure you are asked questions that are relevant to you”. In addition, based on feedback about emotionally triggering parts of the survey, the order in which the GENDER-Q scales appear in the survey was changed with the HRQL scales, including Gender Dysphoria, moved closer to the end of the survey. Participant feedback informed changes to the clinical and demographic questions, including the addition of response options (i.e., I am not sure, prefer not to answer), rewording of hard-to-understand or answer questions (e.g., “Have you ever taken hormones or medication for the purpose of gender affirmation” was changed to “Have you taken hormones or medication in the past that you are no longer taking for the purpose of gender affirmation”), providing definitions (e.g., for inner and outer labia), improving inclusivity (e.g., “chest masculinization” was changed to “chest surgery”), and addition of questions to improve clarity and interpretation of results (e.g., a question about the importance of facial hair and the concern about Adam’s apple).

## Discussion

The field test version of the GENDER-Q comprises 55 independently functioning scales, covering a broad range of concepts across 13 domains relevant to individuals seeking gender-affirming care—HRQL, sexual, urination, gender practices, voice, hair, face and neck, body, breasts, genital feminization, chest, genital masculinization and experience of care. The scales were meticulously designed to enhance patient-centered shared decision-making, advance comparative effectiveness research, and support value-based gender-affirming care.

The GENDER-Q responds effectively to the demand for a rigorous, validated gender-affirming care-specific PROM by the clinical and academic communities [1, 4–8, 18]. The GENDER-Q addresses the limitations of existing PROMs used in TGD research through its adherence to internationally established guidelines for PROM development. The life stories of 84 TGD participants from four countries with different politico-legal and healthcare environments were used to create the GENDER-Q scales to ensure that the scales measured important and relevant outcomes. Additionally, the development process included a large, internationally recognized group of clinical experts in gender-affirming care, several of whom self-identified as TGD. The GENDER-Q program of research embodies a collaborative effort among lived experience experts, clinicians, HRQL researchers and PROM developers, drawing on both experiential and empirical knowledge to enhance its relevance and applicability.

Several pragmatic considerations in the development of the GENDER-Q warrant discussion. The cognitive debriefing interviews conducted to refine the scales were limited to English-speaking participants in Canada and the US. This decision was made to conserve resources related to translating the draft scales into Dutch and Danish and to avoid the need for translating the cognitive debriefing interviews to English for analysis, particularly given that the interviews were conducted in rounds. Further, depending on the scale, 7–14 participants reviewed the draft scales. While this number of patient participants may appear imbalanced compared to the number of clinical experts (ranging from 4 to 37 per scale) providing feedback, the sample size met the recommended sample size criteria for content validity established by the COSMIN guideline [10]. The revisions were made to the GENDER-Q scales in 4 iterative rounds, and it was not feasible to track the reasons for item deletion, addition or revision due to the large number of items tested. Additionally, the sample in part 1 and 2 included few individuals who identified as nonbinary. To enhance the relevance of the GENDER-Q scales across the gender spectrum, we implemented sampling quotas to recruit a larger number of nonbinary participants for the pilot field test. Other limitations of the pilot field test include a lack of diversity in age, race and educational attainment and a lack of participants who had undergone genital gender-affirming surgery. This last limitation hindered our ability to explore the psychometric performance of certain scales prior to the international field test. The international, multi-language field test (step 2) of the GENDER-Q (completed in 2024) overcomes these limitations through the recruitment of a large and diverse international sample from clinical and community settings.

## Conclusions

The GENDER-Q represents the most comprehensive set of scales that are specific to measuring outcomes of gender-affirming care. The international field test was completed in 2024. The GENDER-Q is available for use in patient care, clinical research and quality improvement efforts through www.qportfolio.org.

## Electronic supplementary material

Below is the link to the electronic supplementary material.

Supplementary Material 1

## Data Availability

The datasets used and/or analysed during the current study are available from the corresponding author on reasonable request.

## Abbreviations

HRQL: Health-related quality of life
COSMIN: COnsensus-based Standards for the selection of health Measurement INstruments
PRO: Patient-reported outcome
PROM: Patient-reported outcome measure
RMT: Rasch Measurement Theory
TGD: Transgender and gender diverse
US: United States
USD: United States Dollar
