# Supplementary material for: International study to develop a patient-reported outcome measure to evaluate outcomes of gender-affirming care - the GENDER-Q
Source: J Patient Rep Outcomes. 2024 Nov 19;8:134. doi: 10.1186/s41687-024-00785-x (PMC11576686; doi:10.1186/s41687-024-00785-x)
Supplement: Supplementary file 1 — Supplementary Material 1 [file 41687_2024_785_MOESM1_ESM.docx]

**Supplementary material 1. SEMI-STRUCTURED INTERVIEW GUIDES**

| **Part 1. Evaluating content validity of existing scales**  **Part 3. Scale refinement** |
| --- |
| ***Instructions***   - What are the instructions asking you to do? Please explain in your own words. - Is the time frame for responding to the scale appropriate? - Are there any words we should change to make the instructions easier?   ***Items***   - In your own words, what is this item is asking? - What do you think of when answering this item? - Are any words difficult to understand or offensive? - Was this item hard or easy to answer and why? - Does this item measure an important issue for you? - What do you think about the response choices?   ***At the end of each scale***   - In your own words, what is this group of items asking about? - Does this group of items measure an important issue for you? - Are there any items that do not belong with the rest? - Thinking about that group of items, what are we missing?   ***At the end of the interview***   - What are your overall thoughts about the questionnaire? - Is there anything we forgot to ask that is important to patients seeking treatment? - Is there anything we should change about our questionnaire? - Is there anything else that you would like to add or comment on? |

| **Part 2: concept elicitation for new scales** |
| --- |
| ***Experience of care***   - Can you tell me about the first time you asked for professional help with your gender identity? - Can you tell me about the events that led to your decision to seek treatment? - Have you experienced any barriers to obtaining gender-affirming treatments? - What are the people like who have cared for you? Probe: helpful, friendly, expertise, skill. - What kind of information and advice were you given by healthcare providers? Probe: amount, content, interactions. - How could healthcare professionals improve the quality of care provided to you?   ***Treatments and recovery***   - What kinds of gender-affirming treatments have you had so far? Probe: psychological, hormonal, surgical. - What was the recovery like from surgical treatments? Probe: symptoms and impact on activities and daily life. - What was good/bad about each gender-affirming treatment you have had? - How happy/satisfied are you with each gender-affirming treatment you have had? - Do you plan to have any gender-affirming treatments in the future? If yes, which ones?   ***Appearance***   - How important is your appearance to you? - To what extent is your appearance aligned with your gender identity? - How would you describe your appearance? Probe: face/body/hair/other. - What do you like/dislike about the appearance? Probe: face/body/hair/other. - Has your appearance changed with any gender-affirming treatments and how? Probe: face/body/hair/other. - Is there anything else about your appearance that you would like to change? Probe: face/body/hair/other.   ***Voice***   - How important is how your voice sounds to you? - To what extent is your voice aligned with your gender identity? - How would you describe your voice? - What do you like/dislike about your voice? - How has your voice changed with any gender-affirming treatments and how? Probe: do people respond to you differently?   ***Psychological***   - How do you generally feel? Probe: negative (anxiety, depression, irritation) and positive (happy, post-traumatic growth). - Does distress interfere with daily activities and how? Probe: work, social, dating. - Has your emotional health changed with any gender-affirming treatments and how? - Which gender-affirming treatments have helped the most in terms of emotional well-being?   ***Body image***   - How do you feel about your face/body/hair/other? Probe: negative (self-conscious, uncomfortable, unattractive, abnormal) and positive (confident, attractive, normal). - Do you hide or cover parts of your body or face or modify your voice to blend in? Probe for how and why. - To what extent is your body image in line with your gender identity? - Has your body image changed with any gender-affirming treatments and how? - Which gender-affirming treatments have helped the most in terms of body image?   ***Social***   - What has it been like for you socially? Probe: school, work, friends, family, dating. - Can you describe the kinds of emotional, informational and instrumental support you receive from your social network? - Have you experienced any bullying, teasing, abuse, stigmatisation or discrimination? Probe: school, work, friends, family, dating and coping strategies. - Are there things you want to do in life but do not because of gender-specific issues? - Has your social life changed with any gender-affirming treatments and how? Probe: school, work, friends, family, dating. - Which gender-affirming treatments have helped the most in terms of your social life?   ***Physical***   - To what extent is your physical function aligned with your gender identity? Probe: mobility, strength, activities. - How has your physical function changed with the gender-affirming treatments? Probe: mobility, strength, activities. - Which gender-affirming treatments have helped the most in terms of physical function?   ***Sexual***   - How important is your sexual life and well-being to you? - How do you feel about your body sexually? Probe: positive (confident, attractive, normal) and negatives (self-conscious, uncomfortable, unattractive, abnormal) and clothed/unclothed. - Can you tell me about any problems or concerns with the sexual life related to your gender identity? - In what ways has gender-affirming treatment changed your genitalia? Probe: appearance, function. - Has your sexual life changed with any gender-affirming treatment and how? Probe: frequency, quality, drive, arousal. - Which gender-affirming treatments have helped the most in terms of your sexual life?   ***Additional questions***   - Can you describe any other concerns or issues you experienced that we have not already covered?   Thinking back over this interview, what are the most/least important issues that we have talked about? |

**Supplementary material 2: GENDER-Q FIELD-TEST SCALES AND THE NUMBER OF EXPERTS AND PATIENTS TO PROVIDE FEEDBACK ON EACH SCALE**

| **Domain** | **GENDER-Q Scales** | **Context of use** | **Expert review** | **Patient review** |
| --- | --- | --- | --- | --- |
| HRQL | Body Image | Any treatment | 26 | 14 |
|  | Gender Dysphoria | Any treatment | 26 | 14 |
|  | Social Acceptance | Any treatment | 27 | 14 |
|  | Psychological Distress | Any treatment | 28 | 12 |
|  | Psychological Well-Being | Any treatment | 28 | 12 |
|  | Treatment Outcome | All surgery | 35 | 9 |
| Sexual | Sexual Well-Being | If sexually active, Genital surgery | 26 | 14 |
|  | Orgasm*** | If sexually active, Genital surgery | 0 | 14 |
| Urination | Urination | Any genital surgery | 31 | 7 |
|  | Urinary Catheter | Any genital surgery | 31 | 8 |
| Gender Practices | Binding Well-Being | If binding | 14 | 8 |
|  | Binding: Adverse Effects | If binding | 14 | 14 |
|  | Tucking: Adverse Effects | If tucking | 23 | 7 |
| Voice | Sound | Speech surgery or therapy | 22 | 11 |
|  | Distress | Speech surgery, therapy | 22 | 11 |
| Hair | Hair – Face | Hair removal or addition | 28 | 7 |
|  | Hair – Face^$^ | Hair removal or addition | 28 | 7 |
|  | Hair - Head | Scalp advance, transplant | 27 | 14 |
| Face & Neck | Face | Female feminization | 25 | 7 |
|  | Upper Face | Female feminization | 25 | 7 |
|  | Nose | Rhinoplasty | 26 | 7 |
|  | Nostrils | Rhinoplasty | 25 | 7 |
|  | Lips | Surgery or filler | 25 | 7 |
|  | Cheeks** | Implant, fat graft | 16 | 7 |
|  | Chin | Surgery, implant | 25 | 7 |
|  | Jawline | Surgery, shave | 25 | 7 |
|  | Adam’s Apple | Tracheal shave | 26 | 7 |
| Body | Body** | Contouring, liposuction | 15 | 7 |
|  | Buttocks** | Contouring, liposuction | 14 | 7 |
|  | Waist** | Contouring, liposuction | 14 | 7 |
| Breasts | Breasts | Feminizing top surgery, fat grafting | 28 | 7 |
|  | Nipples Areolas | Masculinizing top surgery, fat grafting | 29 | 7 |
|  | Animation Deformity** | Submuscular implants | 20 | 7 |
| Genital Feminization | Vagina | Vaginoplasty | 29 | 7 |
|  | Labia | Labiaplasty | 29 | 7 |
|  | Clitoris | Feminizing genital surgery | 29 | 7 |
| Chest | Chest | Masculinizing top surgery | 30 | 7 |
|  | Nipples/areolas | Masculinizing top surgery, fat grafting | 29 | 7 |
|  | Scars** | Masculinizing top surgery | 19 | 7 |
| Genital Masculinization | Penis | Phalloplasty, Metoidioplasty | 31 | 7 |
|  | Penis Sensation | Phalloplasty, Metoidioplasty | 32 | 7 |
|  | Glans | Glansplasty | 28 | 7 |
|  | Scrotum | Scrotoplasty | 29 | 7 |
|  | Perineum | Surgery to create perineum | 29 | 7 |
|  | Phalloplasty Flap | Phalloplasty | 29 | 7 |
|  | Phalloplasty Scars** | Phalloplasty | 29 | 7 |
|  | Donor Site: Adverse Effects | Phalloplasty | 29 | 7 |
|  | Testicular implants | Phalloplasty, Metoidioplasty | 29 | 7 |
|  | Erectile device | Phalloplasty | 29 | 7 |
| Experience of care | Healthcare Professional | Quality improvement | 37 | 11 |
|  | Clinic | Quality improvement | 37 | 10 |
|  | Surgery - Information | Quality improvement | 35 | 10 |
|  | Surgery - Adverse Effects | Any surgery | 31 | 12 |
|  | Surgery - Return to Activity | Any surgery | 30 | 14 |

**Scales added after round 1 of scale refinement and reviewed by up to 26 experts.

***This scale was only reviewed by patients
